# Dynamic Rheological Studies of Poly(*p*-phenyleneterephthalamide) and Carbon Nanotube Blends in Sulfuric Acid

**DOI:** 10.3390/ijms11041352

**Published:** 2010-03-31

**Authors:** Yutong Cao, Zhaofeng Liu, Xianghua Gao, Junrong Yu, Zuming Hu, Ziqi Liang

**Affiliations:** 1 State Key Laboratory for Modification of Chemical Fibers and Polymer Materials, Donghua University, Shanghai 201620, China; 2 National Renewable Energy Laboratory, Golden, CO 80401, USA

**Keywords:** liquid crystalline (LC) solution, single-walled carbon nanotubes (SWNTs), rheology

## Abstract

We have studied the dynamic scanning of liquid-crystalline (LC) poly(*p*-phenyleneterephthalamide) sulfuric acid (PPTA-H_2_SO_4_) solution, and its blend with single-walled carbon nanotubes (SWNTs), by using a flat plate rotational rheometer. The effects of weight concentration and molecular weight of PPTA, as well as operating temperature, on dynamic viscoelasticity of the PPTA-H_2_SO_4_ LC solution system are discussed. The transition from a biphasic system to a single-phase LC occurs in the weight concentration range of SWNTs from 0.1% to 0.2%, in which complex viscosity reaches the maximum at 0.2 wt% and the minimum at 0.1 wt%, respectively, of SWNTs. With increasing SWNT weight concentration, the endothermic peak temperature increases from 73.6 to 79.9 °C. The PPTA/SWNT/H_2_SO_4_ solution is in its plateau zone and storage modulus (*G*′) is a dominant factor within the frequency (ω) range of 0.1–10 rad/s. As ω increases, the *G*′ rises slightly, in direct proportion to the ω. The loss modulus (*G*″) does not rise as a function of ω when ω < 1 s^−1^, then when ω > 1 s^−1^ G″ increases faster than *G*′, yet not in any proportion to the ω.

## 1. Introduction

**Scheme 1.**
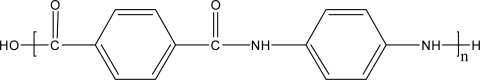
Molecular structure of poly(*p*-phenylenetetraphthalamide) (PPTA).

A liquid-crystalline (LC) solution is an orientationally ordered liquid that displays fluidic characteristics of the liquid state while the molecules still maintain a directional order. There are three main classes of LC phases, *i.e.*, nematic, smectic and cholesteric phase, which were originally identified by Friedel *et al.* according to the degree of order and molecular alignment of each phase [1]. Poly(*p*-phenyleneterephthalamide) (PPTA), a rigid-rod polymer as shown in Scheme 1, is typical of high tensile strength, remarkable stiffness, and excellent thermal stability [2]. One of the most interesting physical properties of PPTA macromolecules is their strong affinity to self-organize in a suitable solvent such as absolute sulfuric acid (H_2_SO_4_) and hence form LC phases [3]. Such strong molecular arrangement is attributed to the extreme anisometry of rigid PPTA and disruption of intermolecular hydrogen bonds in PPTA caused by the super-acidity of H_2_SO_4_. Nematic sulfuric acid LC solution of PPTA, referred to as PPTA-H_2_SO_4_, with a weight concentration range of 19–20% when heated to 80–95 °C has been used to fabricate high-strength fibers by dry-jet wet-spinning process [4–6]. The unusual rheology phenomena of PPTA-H_2_SO_4_ LC solution have been reported [7–11], including strong shear-thinning, long relaxation time, and high viscous activation energy. These notable behaviors inspired us to investigate the dynamic rheological properties of the PPTA-H_2_SO_4_ LC solution system in the spinning process. We then carried out the dynamic scanning of the PPTA-H_2_SO_4_ LC solution system with a flat plate rotational rheometer and discussed the effects of weight concentration and molecular weight of PPTA, as well as temperature, on such viscoelastic properties as complex viscosity (η^*^), storage modulus (*G*′), loss modulus (*G*″), and loss-tangent (*tanδ*). Our results shown in this paper provide some general fundamental insight for the LC spinning process.

Single-walled carbon nanotubes (SWNTs) have been shown to possess extraordinary mechanical, electrical and thermal properties [12–15]. Polymers have been shown to be significantly reinforced with SWNTs [16,17], and some typical examples include pitch-based/SWNT composite carbon fibers [18], PBO/SWNT [19], and polyacrylonitrile/SWNT composite fibers [20]. The major challenge, however, remains to achieve uniform and stable dispersions of SWNTs in the polymer matrix with significant nanotube weight-fractions. It is known that SWNTs can be successfully dispersed in sulfuric acid. The nematic structure of SWNT/H_2_SO_4_ solution has been evidenced by birefringence and rheological curves [21]. It is thus of special interest to investigate whether rigid rod-like polymers (e.g., PPTA) can be reinforced with SWNTs in a large loading ratio, both in the LC solution, which will be of great significance to wet-spinning production of high-performance fibers. For this purpose, LC solution of PPTA/SWNT/H_2_SO_4_ was prepared, and its thermal and rheological behaviors were studied accordingly. Our study aims to assess the effects of SWNTs on the properties of the spinning solution. Through our studies, we are able to identify the optimal range of SWNTs concentration and spinning temperature for dry-jet wet-spinning process of PPTA fibers.

## 2. Results and Discussion

### 2.1. Effect of Weight Concentration of PPTA on Rheological Behaviors

**Figure 1.**
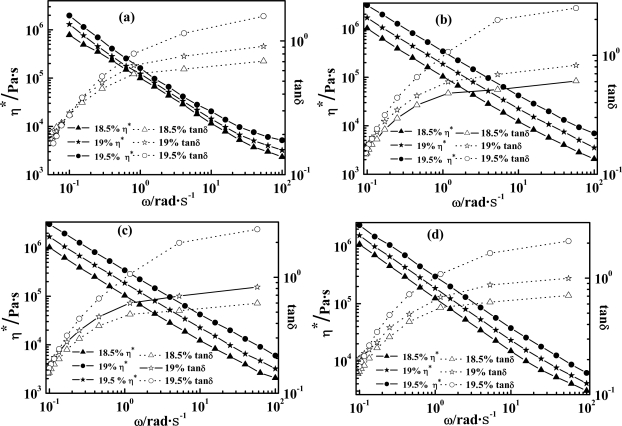

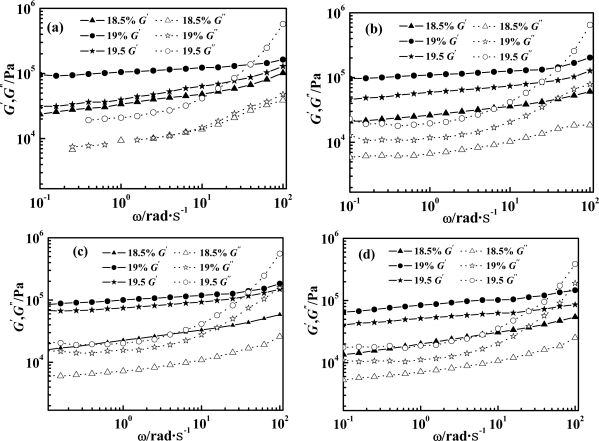
Rheological curves dependences of PPTA-H_2_SO_4_ LC solution on shearing frequency (*ω*), PPTA wt% at 18.5, 19 and 19.5%, and temperatures of (**a**) 75 °C, (**b**) 80 °C, (**c**) 85 °C and (**d**) 90 °C.

Effects of the variation of weight concentrations of PPTA (PPTA wt%: 18.5, 19 and 19.5%) and temperatures (75, 80, 85, 90 °C) on rheological curves of the PPTA-H_2_SO_4_ LC solution are shown in Figure 1. The solution exhibits a typical shear-thinning behavior even at a low shearing frequency (*ω*), *i.e.*, its η^*^ decreases as *ω* increases, which results in the alignment of PPTA chains in parallel. With increasing *ω*, the density of junction points decreases since the crosslinks break significantly faster than they form owing to their shear-thinning behavior [22]. Such changes of chain configuration and orientation under shearing force indicate that both *G*″ and *tanδ* of PPTA-H_2_SO_4_ LC solution would increase as *ω* rises. At a high shearing rate, nearly all macromolecular chains orient, implying that *G*′ hardly follows the variation of frequency, since in this regard the first normal stress difference hardly follows frequency variation [23].However, when weight concentration of the solution increases, density of entanglement crosslinks and intermolecular forces both increase, and then macromolecular chains become more difficult to orient and diffuse, leading to the increase in all the rheological characteristics including η^*^, *G*′, *G*″, and *tanδ*. On the other hand, as the temperature increases, thermal motion of macromolecular chains increases, flow resistance decreases, and rheological curves of η^*^, *G*′, *G*″, and *tanδ* move down.

### 2.2. Effect of Molecular Weight of PPTA on Rheological Behaviors

**Figure 2.**
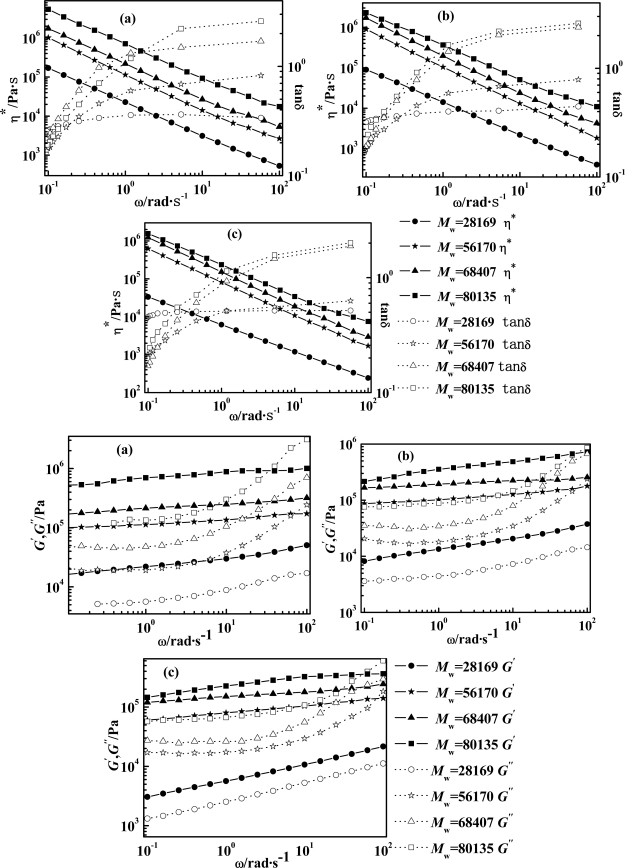
Effect of PPTA molecular weight of PPTA-H_2_SO_4_ LC solution on rheological curves *versus* shearing frequency (*ω*).

Supramolecular structure is key to affecting the rheological characterization of the polymer system. Figure 2 displays the rheological behaviours of PPTA-H_2_SO_4_ LC solution at 19.5 wt% of PPTA as a function of *M_w_* (*i.e.*, 28169, 56170, 68407, and 80135 g/mol) and temperature (*i.e.*, 80, 85, and 90 °C). All four rheological parameters (η^*^, *G*′, *G*″, and *tanδ*) increase with increasing *M_w_* of polymers. Also, the larger *M_w_* is, the longer relaxation time (λ) appears, meaning that oriented molecular chains upon shearing cannot resile, thus showing more distinct shear-thinning behaviour. This is greatly advantageous to obtain high degree of orientation in spinning process and hence obtain fibers with higher tenacity and modulus. Moreover, the larger *M_w_* is, the more entanglement crosslinks are generated, meaning that the dynamic process of disentanglement and entanglement would again take place at less shear rate [24,25]. Hence, *M_w_* of PPTA would be ideally neither high (difficult for molding) nor low (accelerating the relaxation).

### 2.3. Effect of Weight Concentration of SWNT on Rheological Behaviors

**Figure 3.**
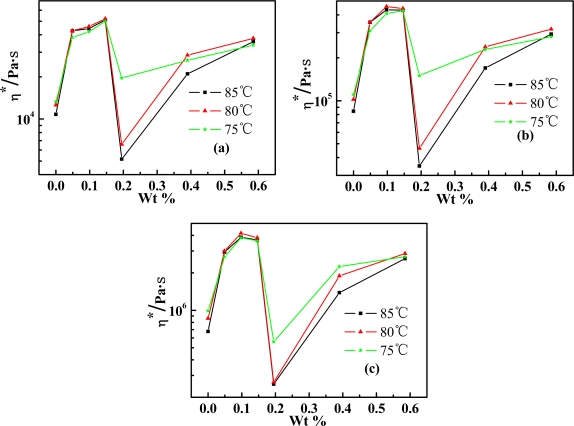
Dependence of complex viscosity (η^*^) of PPTA/SWNT/H_2_SO_4_ dopes on temperature and SWNT wt% at a shear frequency (*ω*) of (**a**) 10 s^−1^, (**b**) 1 s^−1^ and (**c**) 0.1 s^−1^.

Variation of complex viscosity as a function of SWNT weight concentration (SWNT wt%) in PPTA/SWNT/H_2_SO_4_ dopes is plotted in Figure 3, showing the nonmonotonic characteristic as a function of frequency and temperature. The η^*^ first gradually increases with increasing SWNT wt%, and then decreases abruptly when beyond ∼0.1 SWNT wt%, and arises again when beyond 0.2 SWNT wt% to reach about the same viscosity for three temperature-dependent curves. In general, this concentration dependent viscosity behavior of PPTA/SWNT/H_2_SO_4_ solution resembles that of SWNT/H_2_SO_4_ solution. As the SWNT concentration was below 0.1 wt%, the solution viscosity increased accordingly with increasing SWNT wt%. This can be considered as normal behavior of an isotropic solution. When SWNT concentration was exceeded 0.1 wt%, the viscosity decrease at increasing SWNT concentration can be considered as a unique behavior of anisotropic solution. It is caused by the liquid crystal behavior where SWNT are aligned upon shear and form domains of highly ordered structures.

It has been revealed in rheology and microscopy studies that SWNTs in H_2_SO_4_ exhibit roughly similar phase behavior as rod-like polymer solutions [21,26]. We have shown in optical microscopy studies that SWNTs in sulfuric acid solution tend to self-assemble into extremely long strandlike structures, which is typically characteristic of the LC phase [21]. The LC transition from a biphasic phase to a single phase occurs in the SWNT wt% range from 2.7 wt% (maximum viscosity) to 4 wt% (minimum viscosity) [21]. For a blend of SWNT with such a rigid-rod polymer as PPTA, SWNT appears easier to adopt a higher LC order as the transition occurs within the lower SWNT wt% range [27,28]. Thus, 0.1 wt% was the critical concentration of SWNT for PPTA/SWNT/H_2_SO_4_ system. A biphasic region of mixed isotropic and anisotropic phase existed between 0.1 wt% and 0.2 wt%. Beyond 0.2 wt%, the solution became an increasingly anisotropic-rich phase, and the solution viscosity increased again with increasing SWNT wt%.

### 2.4. Temperature Dependence of Rheological Behaviors

**Figure 4.**
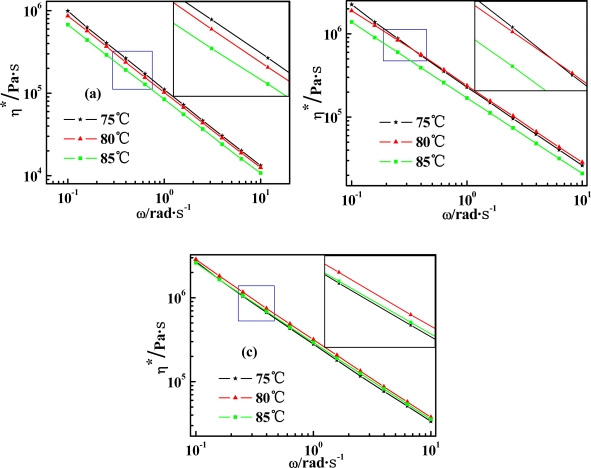
Temperature dependence of complex viscosity (η^*^) of (**a**) PPTA/H_2_SO_4_, (**b**) PPTA/SWNT (98/2 wt%)/H_2_SO_4_, and (**c**) PPTA/SWNT (97/3 wt%)/H_2_SO_4_ as a function of shearing frequency (*ω*). PPTA is kept at 19.5 wt% in all above cases.

Temperature effect on complex viscosity (η^*^) as opposed to shearing frequency (*ω)* of PPTA/SWNT/H_2_SO_4_ dope is shown in Figure 4.

All three samples composed of pure PPTA and PPTA coupled with different portion of SWNT exhibit an obvious shear-thinning behavior through the entire *ω* range. As *ω* increases, η^*^ decreases in direct proportion to *ω*. The slope of such decreasing line on the logarithmic plot remains similar, at about −0.91, for all three samples. This shear-thinning behavior is thought to arise from entanglements between macromolecular chains. For rigid-rod polymers such as PPTA, shearing decreases the number of entanglements and thus affords macromolecules to rearrange the configurations readily. By comparison, PPTA/SWNT/H_2_SO_4_ dopes are more highly shear-sensitive. (1) The η^*^ of PPTA/H_2_SO_4_ dope decreases as temperature increases from 75 to 85 °C. Such behavior is often observed among conventional polymers. (2) The η^*^ of PPTA/SWNT (98/2 wt%)/H_2_SO_4_ dope decreases as temperature increases from 80 to 85 °C, while it becomes nearly independent of temperature ranging between 75 °C and 80 °C. (3) The η^*^ of PPTA/SWNT (97/3 wt%)/H_2_SO_4_ dope decreases slightly as temperature increases from 80 to 85 °C, but increases slightly as temperature increases from 75 to 80 °C.

**Figure 5.**
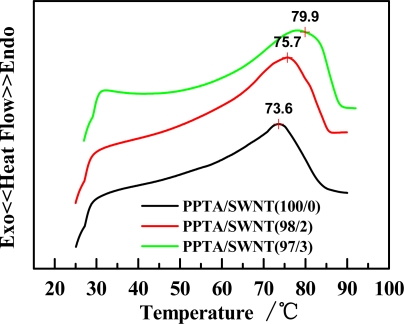
DSC heating curves of PPTA/SWNT/H_2_SO_4_ dope with different weight ration of PPTA and SWNT.

To determine possible phase changes from 75 to 85 °C, we carried out thermal analysis on PPTA/SWNT/H_2_SO_4_ dopes. The DSC thermogram (Figure 5) reveals a broad endothermic peak for all samples, implying that multiple transitions occur including monothetic to the liquid state and also isotropic to nematic phase [25,29]. We also noted that with increasing SWNT wt%, the endothermic peak temperature increases from 73.6 to 79.9 °C, possibly because the curve of η^*^ vs. SWNT wt% becomes nearly independent of temperature [30].

**Figure 6.**
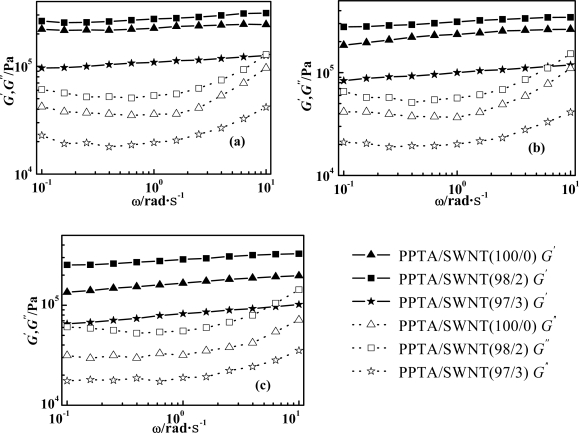
Dynamic modulus (*G*′ and *G*″) of PPTA/SWNT/H_2_SO_4_ dope as a function of shearing frequency (*ω*) at temperatures of (**a**) 75 °C, (**b**) 80 °C, and (**c**) 85 °C.

Figure 6 presents the temperature dependent plots of the dynamic modulus of PPTA/SWNT/H_2_SO_4_ dopes over *ω*. As SWNT wt% increases, *G*′ and *G*″ arise sharply regardless of temperature. For any temperature and SWNT wt%, elastic characteristic such as *G*′ of PPTA/SWNT/H_2_SO_4_ dope is a dominant factor within the frequency range from 0.1 to 10 rad/s. As *ω* increases, the *G*′ rises slightly in direct proportion to *ω*. The slope of *G*′ *versus* *ω* on the logarithmic plot is 0.098, 0.085 and 0.063 as PPTA/SWNT is 100/0, 98/2 and 97/3, respectively. In a low *ω* region (*ω* < 1 s^−1^), the *G*″ does not rise, while in other regions (*ω* > 1 s^−1^), *G*″ rises faster than *G*′, but not in proportion to frequency.

According to the relative magnitude of *G*′ and *G*″, a logarithmic plot of *G*′ and *G*″ as a function of frequency comprising three zones: terminal, plateau and transition zone, has been proposed [31]. Referred to the literature, PPTA/SWNT/H_2_SO_4_ dope is in its plateau zone within the frequency range from 0.1 to 10 rad/s [32]. Rod-like macromolecules like PPTA are thought to exhibit longer relaxation times than flexible polymers due to the difficulty of molecular reorientation arising from the stiff molecular structure and close-packing. The dynamic behavior of Vectra^®^, for example, supports this argument [33]. As SWNT wt% increases, PPTA/SWNT/H_2_SO_4_ dope seems to exhibit more intermolecular interaction and chain rigidity, thus giving rise to the plateau-zone behavior [34].

## 3. Experimental Section

### 3.1. Materials and Preparation

Sulfuric acid with 0.2 wt% excess SO_3_ was prepared by mixing the fuming sulfuric acid (25 wt%) with concentrated sulfuric acid (98 wt%). The concentration of sulfuric acid was measured by acid-base titration. The SWNTs used in this study was acquired from Shenzhen Nanotechnologies Co., Ltd. and an average diameter of <2 nm. Finally, SWNTs were dried under vacuum overnight at 80–100 °C. Dispersions were prepared by mixing with a magnetic stir bar for 3 days in an anhydrous environment in a glovebox which was nitrogen atmosphere at room temperature [35]. The PPTA polymer was synthesized following the literature methods [36,37]. All glasswares were dried before use.

The PPTA-H_2_SO_4_ LC solutions were prepared with four different molecular weight polymers *(M_w_* = 28169, 56170, 68407, 80135 g/mol), at different temperatures (75, 80, 85, 90 °C) and with different weight concentrations (18.5, 19, 19.5 wt%). For example, into a 250 mL three-neck flask equipped with a mechanical stirrer and a nitrogen inlet/outlet, were placed 34.01 g of PPTA and 140.4 g of H_2_SO_4_ at 75 °C. The mixture was then stirred for 2.5 h and opalescence was observed during this step. Likewise, the LC solutions of PPTA/SWNT/H_2_SO_4_, in which PPTA was kept at 19.5 wt% and PPTA/SWNT is 99/1 wt%, were prepared in a 250 mL three-neck flask. SWNTs (0.3401 g) were dispersed in H_2_SO_4_ (140.4 g) under an inert and anhydrous atmosphere with mechanical stirring and at room temperature for at least 3 days [21]. Into this flask were then added 34.01 g of PPTA at 80 °C. The mixture was stirred for 2.5 h and stir opalescence was observed during this step.

### 3.2. Rheological Properties

Subsequently, the PPTA-H_2_SO_4_ LC and PPTA/SWNT/H_2_SO_4_ dope solutions were performed with dynamical scanning on an ARES rotational rheometer (TA Instruments, New Castle, DE, USA) at four temperatures (75, 80, 85, 90 °C) in the frequency range of 0.01–100 rad·s^−1^ and 0.1–10 rad·s^−1^, respectively. Testing fixtures parallel plates (50 mm) were made of stainless steel 316 to avoid corrosion. In order to prevent water exchange between the sample and environment, the sample surface between the plate and the cone was coated with a mineral oil [38,39]. The effects of dynamic frequency (*ω*) on rheological behaviors such as η^*^, *G*′, *G*″ and *tanδ* have been examined.

### 3.3. Characterization

Thermal analysis on the PPTA/SWNT/H_2_SO_4_ dope was carried out on a differential scanning calorimeter (DSC) (Mettler Toledo 822) under a N_2_ atmosphere at a heating rate of 5 °C/min ranging from 25 to 90 °C.

## 4. Conclusions

We have shown that PPTA-H_2_SO_4_ LC solution exhibits a typical shear-thinning behavior. Our rheology studies are summarized as follows. (1) As PPTA wt% increases, rheological curves of η^*^, *G*′, *G*″ and *tanδ* all move up. By contrast; (2) as temperature rises, rheological curves of η^*^, *G*′, *G*″ and *tanδ* all move down. Finally, (3) η^*^, *G*′, *G*″ and *tanδ* all increase as *M_w_* of PTTA increase.

When SWNTs are added into PPTA-H_2_SO_4_ LC solution, the protonation of SWNTs in superacids enables their good dispersion to form homogeneous PPTA/SWNT/H_2_SO_4_ dope solution. The dope solution exhibits a remarkable nematic LC phase. Our findings are multi-folds. (1) For all temperature and concentration of SWNT, the complex viscosity of PPTA/SWNT/H_2_SO_4_ dope shows an obvious shear-thinning behavior, and the slope of complex viscosity *versus* frequency on a logarithmic plot is about the identical at *ca.* −0.91. The DSC thermogram displays multiple phase transitions between monotetic and the liquid state, as well as isotropic and nematic phase. As concentration of SWNT increase, *G*′ and *G*″ rise sharply at various temperatures. (2) For all temperature and concentration of SWNT, the elastic characteristic such as *G*′ of PPTA/SWNT/H_2_SO_4_ dope is a dominant factor within the frequency range from 0.1 to 10 rad/s. At sufficiently high concentrations of SWNT, approximately 0.2 wt%, and at 85 °C, a novel type of single-phase nematic liquid crystal is formed. This allows the dope solution to produce superior-performance fibers by dry-jet wet-spinning process.
